# Typho-Malarial Fever, Complicated with Symptoms of Cerebro-Spinal Irritation

**Published:** 1871-10

**Authors:** N. S. Davis

**Affiliations:** Chicago


### TYPHO-MALARIAL FEVER, COMPLICATED WITH SYMPTOMS OF CEREBRO-SPINAL IRRITATION.

Prepared for the Chicago Medical Society, by N. S, DAVIS, M. D., Chicago.

A few weeks since I called attention of the society to several cases of what was regarded as typho-malarial fever, complicated with severe dysentery. Those cases occurred during the month of June, and mostly in a particular part of the south-western portion of the city. From four to six weeks later in the season we began to meet with a class of cases presenting a different, though not much less formidable complication. The cases now alluded to were confined to no one section of the city, although they occurred almost exclusively among the laboring classes and in crowded dwellings. The first that attracted my attention strongly was that of Mr. B —, a laborer, living at 123 Burnside street. He was attacked in the evening of the 20th of July, with a chill accompanied by severe pain in the head, especially in the occipital region and in the cervical and dorsal portions of the spine. I was called to see him on the 21st and found him with an active general fever, skin hot and dry; face flushed; tongue covered with a white fur; pupils natural, but eyes rather sensitive to light; pulse 105 per minute; respiration hurried; and complaining of intense pain in his head and back, aggravated by motion, with hyperæsthesia over the whole length of the spine and lower extremities. There was also some rigidity of the muscles of the spine and extremities. At first I regarded the attack as one of direct inflammation of the cervical and dorsal portions of the spine, probably of a rheumatic character; and he was directed to take small alterative doses of calomel with Dover's powder and nitrate of potassa every four hours, alternately with a mixture of

℞ Tinct. Cimicifuga. ℥ii.

Tinct. Strammonii, ℥ss.

Vin. Clochici Sem, ℥ss.

Simple Syrup, ℥j.

Acetis Potassa, ʒiv.

M. Dose pt. ʒi. in water every 4 hours.

Also a strongly anodyne liniment to the whole length of the spine.

The following day the calomel powders were omitted and a saline laxative given sufficient to evacuate the bowels, the mixture in the vial being continued On the 23d the pains in head and back were less and there was less hyperæsthesia of the extremities, though there remained rigidity of the muscles of the neck, and increase of pain on motion. The general febrile action was less and it had been observed to be distinctly remittent in its charaeter; the exacerbations commencing about 10 o'clock A. M., and declining at midnight. In consequence of this, he was directed to take five grains of sulphate quinine and one quarter of a grain of sulphate of morphia three times during the period of remission each day, and to continue the cimicifuga mixture every four hours. Under this treatment the exacerbations diminished and he seemed so far improved that on the morning of the 30th he got up and shaved himself. But during the afternoon the febrile exacerbation returned with all the cerebro-spinal symytoms, as well marked as at first. But now the lower extremities were in a state of anæsthesia and motionless; the muscles of the neck rigid; the pupils dilated; and the pulse small, somewhat irregular and slow.

He was put on five grain doses of iodide potassa, with 12 drops of tincture of belladonna every two hours; a blister applied to the back of the neck, and 10 grains of quinine ordered to be taken at 9 o'clock P. M. and at 8 o'clock A. M. On the 31st the febrile phenomena were less, but deglutition was difficult and no improvements in the other symptoms. The iodide and belladonna were continued every three hours, and strychnia in doses of one-fortieth of a grain given alternately. The patient continued to fail, the muscles of deglutition, as well as those of the lower extremities becoming entirely paralyzed. On the morning of August 2d, all the muscles of the deglutition and respiration were found paralyzed, and his discharges involuntary. The air passages rapidly filled with mucous which the patient had no power to dislodge, and he expired on the evening of the same day No post mortem examination was allowed.

Mr. R—, a grocer, living in the rear of his grocery at 793 State street, was attacked on the 3d of August. I saw him the following morning, Aug. 4th, and found him presenting almost the same assemblage of symptoms as the case just detailed, only less pain and hyperæsthesia in the spine and lower extremities. The pain in the occiput and through the base of the brain was severe; the muscles of the neck rigid; the pupils of unusual size, and vision indistinct; respiration hurried, at times panting; pulse not over 90 per minute, soft and regular; skin hot; countenance dull, and face suffused with a dark flush. This patient grew steadily worse until the morning of the fourth day, when I put him on the use of fifteen grains of sulphate of soda and fifteen drops of tincture of belladonna every two hours, with a single dose of sulphate of quinine at the time of the greatest remission. From this time the patient began slowly to improve. The same medicine was continued only lengthening the interval between the doses as the cerebro-spinal symptoms abated; and on the 12th of August he appeared convalescent. But the recovery of strength and steadiness in walking was very slow, not being complete in less than five or six weeks.

C. D——, a laborer, aged 23 years, living at No. 83 West Indiana street, was attacked with a severe chill, followed by fever, on the 18th of August, 1871. On the following day the chill was repeated, and I saw him a few hours after. His face was deeply flushed; the skin generally redder than natural; the expression anxious and suffering; eyes watering, but pupils natural; skin hot and dry; pulse 90 per minute; respiration variable, short and about 28 per minute; tongue covered with a thick white coat, but not dry; urine scanty and red; considerable thirst, and stomach rejecting its contents when over-full with drink; bowels quiet, though they had been freely moved by physic the preceding day.

But the most noticeable symptom was intolerable pain in his head, most severe through the region of the base of the brain, from the lower part of the frontal to the occipital region. The head was thrown a little back and the muscles of the neck slightly rigid, with tenderness to the touch. The pain was so severe as to induce a constant moaning and begging for relief. It was stated that these same symptoms had occurred after the chill of the preceding day, lasting six or eight hours, then abating much in severity, but not wholly disappearing until the chill of to-day. Regarding the case as one of periodical fever, accompanied by unusual irritation of the cerebro-spinal nervous centre, he was directed to take twenty grains of bromide potassium, and eight drops of tincture of gelseminum, every hour, until the violence of the paroxysm and the cephalalgia had abated; then to take five grains of sulphate quinine, and two grains of calomel, every three hours, until three doses had been taken. The directions were obeyed, but on visiting the patient the following day, there was found no amelioration of his condition. The febrile exacerbation returned at the usual time, and with even more severe cephalalgia than before. The muscles of the neck were more rigid, and with each expiration there was a well-marked spasmodic jerk in the diaphragm and abdominal muscles.

If there was any modification of the phenomena, it was in the less marked cold stage. I now directed solution of sulphite of soda and tincture belladonna, in such proportion that the patient could take fifteen grains of the first, and fifteen drops of the last, every three hours, without reference to exacerbations or remissions.

For immediate relief of pain, he was directed twenty grains of bromide potassium, and twenty of hydrate of chloral, to be taken at once, and repeated in two hours, if the pain was not somewhat relieved; and twenty grains of quinine, divided into three doses, to be given during the next remission. On calling the next day, August 20th, it was stated that the bromide and hydrate of chloral appeared to have no other effect than to stupify and confuse the mental faculties, without inducing sleep or shortening the paroxysm. There had been some nausea, and some of the medicines had probably been rejected by vomiting. Yet he was found in the midst of another exacerbation, if possible more aggravated, both in the pain and in the derangement of muscular action, than on either of the preceding days. For the purpose of allaying the gastric irritation, and allowing the patient to become entirely free from the influence of the foregoing medicines, he was directed the following solution:

| | | |
|---|---|---|
| ℞ | Carbolic Acid Crystals, | 8 grs. |
| | Glycerine, | ℥ss. |
| | Tinct. Gelseminum, | ℥ss. |
| | Water, | ℥iij. |

Mix. Give one teaspoonful every three hours, in a tablespoon-of water.

August 21st. Visited the patient at the usual hour, about 4 P. M., and found him again in the midst of one of his violent paroxysms of sufferings. His head was drawn back, muscles of the neck quite rigid, and sensitive to the touch; moaning loudly with pain, but indisposed to speak or move; pupils large; countenance dull; temperature of the surface but little above natural; pulse only 85 per minute, soft, and regular; respiration hurried, irregular, and each expiration accompanied by spasmodic action of the diaphragm and abdominal muscles; abdomen empty, concave, but hard from rigidity of muscles; urine scanty. The tongue was still coated, but moist. The bowels had moved once, nearly natural; no vomiting; and during the preceding night the patient had slept quietly for three or four hours.

The posterior cervical muscles had not been entirely free from stiffness, nor the head wholly free from pain, since the commencement of his sickness. Seeing the dilated condition of the pupils unaccompanied by other symptoms of cerebral effusion, and remembering the effects of the Calabar bean in some other cases of irritation of the cerebro-spinal axis, I determined to use it in this case. Accordingly I directed twenty drops of a reliable tincture of Calabar bean to be taken, at first, every two hours, and three grains of quinine, and the one-sixth of a grain of morphine, to be given morning, noon, and night. These directions were carried out, and the next day I found my patient decidedly better. He had an exacerbation at the usual time, but with much less severity.

The treatment was continued, except the interval between the doses of the tincture of Calabar bean was lengthened to three hours. At this rate both remedies were continued through the 22d, 23d, and 24th, the exacerbations becoming less each day, until on the latter day they were hardly observable, and the patient moved his head pretty freely.

After this the quinine and morphine were limited to one dose every night, and the Calabar bean given every six hours. Under this treatment, the patient became entirely convalescent by the 4th of September, and has remained well since.

On the 27th of August, I was called to Miss B., a young woman, on Burnside street, near Twenty-First street. She was found laboring under an attack, in all respects closely resembling the one just detailed. After giving her a few grains of calomel, and opening the bowels, I put her at once on doses of sulphate of quinine three grains, with sulphate morphine one-sixth of a grain, three times a day, and twenty drops of the Calabar bean every two hours the first day, and every three hours subsequently. The symptoms and exacerbations began to lessen visibly within the first twenty-four hours; and in ten days the patient was fully convalescent, having taken no other medicines, except castor oil enough, twice, to move the bowels. A few other cases, of less severity, have since been treated with these remedies. And I am satisfied that in some forms of irritation, or morbid excitement of the cerebro-spinal nervous system, especially accompanied by muscular rigidity, we may find the Calabar bean a valuable remedy.

